# Supplementary material for: Chinese Consumers’ Trust in Food Safety Surveillance Sampling of Commonly Consumed Foods
Source: Foods. 2022 Jul 2;11(13):1971. doi: 10.3390/foods11131971 (PMC9265750; doi:10.3390/foods11131971)
Supplement: Supplementary file 1 [file foods-11-01971-s001.zip › foods-1764382-SI.pdf]

foods MDPI

Supplementary file 1 Definitions

Commonly consumed foods: The State Administration of Market Supervision and Administration of the People's Republic of China, in its Notice on Food Safety Supervision Sampling Inspection by Market Supervision Departments, mentions six categories of "commonly consumed" products: edible agricultural products, processed grain products, meat products, edible oils/grease products, dairy products, and egg products. In this study, these six food groups were defined as commonly consumed foods.

Food safety supervision sampling inspection: The sampling, inspection, re-inspection, and disposal activities of food products organized by market supervision and administration departments according to legal procedures, food safety standards, and other regulations for food risk screening.

The qualified rate of safety supervision sampling inspection of commonly consumed foods: The rate of the number of samples or batches that qualifies in the sampling inspection relative to the total number of samples or batches sampled during the supervision sampling inspection of commonly consumed foods; expressed as a percentage.

Supplementary file 2 Questionnaires

1. Socio-demographic characteristics

Gender ☐Male ☐Female

Age ☐<30 ☐30–39 ☐40–49 ☐50–59 ☐≥60

Education level

☐Junior high school degree or below ☐Senior high school degree

☐College degree ☐Bachelor's degree or above

Annual per-capita household income

☐<10,000 ☐10,000–19,999 ☐20,000–29,999

☐30,000–39,999 ☐40,000–59,999 ☐≥60,000

Place of residence ☐urban ☐rural

You are mainly responsible for food purchasing/cooking in your household ☐Yes ☐No

2. Trust in supervision sampling inspection

(From 1 to 5: 1=strongly disagree; 2=disagree; 3=not sure; 4=agree; 5=strongly agree.)

I think that the government regulators have good expertise in food safety supervision sampling inspection (e.g., can accurately understand the status of food safety in the country and identify food safety hazards).

I think that the government supervision department is extremely good at food safety supervision sampling inspection.

I think that the government regulators have a strong scientific approach to food safety supervision sampling inspection.

Government regulators can be fair and just in terms of food safety supervision sampling inspection.

Government regulators can be honest and not conceal anything from the public in food safety supervision sampling inspection.

Government regulators can fully consider and safeguard the public interest in food safety supervision sampling inspection.

3. Generalized Trust

(From 1 to 5: 1=strongly disagree; 2=disagree; 3=not sure; 4=agree; 5=strongly agree.)

I think government departments can be trusted.
I think most strangers are trustworthy.

4. Perception of food safety

(From 1 to 5: 1=strongly disagree; 2=disagree; 3=not sure; 4=agree; 5=strongly agree.)

Compared to five years ago, food has become safer.
In the past five years, the overall food safety situation in China has remained stable.

5. Attitude toward high qualified rate of supervision sampling inspection

(From 1 to 5: 1=strongly disagree; 2=disagree; 3=not sure; 4=agree; 5=strongly agree.)

I agree with the results of the high qualified rate for supervision sampling inspection.
I support the results of the high qualified rate for supervision sampling inspection.
I believe that the results of the high qualified rate are consistent with the actual food safety situation.
I am satisfied with the results of the high qualified rate for supervision sampling inspection.

6. Improvement measures for food safety supervision sampling inspection

(From 1 to 5: 1=strongly disagree; 2=disagree; 3=not sure; 4=agree; 5=strongly agree.)

The sampling scheme should be scientific and reasonable (e.g., focusing on key issues, key varieties, large enterprises, and small workshops).
Sampling process should be open and transparent.
Sampling and analysis techniques should be accurate.
Sampling and testing results should be open and transparent.
Sampling and testing results should be interpreted in detail to respond to public concerns.
Substandard products should be effectively traced and recalled.
The most stringent standards should be established.
Rigorous regulation should be implemented.
The most severe penalties should be imposed.
The most serious accountability should be upheld.

Supplementary file 3

Table S1. Comparison of socio-demographic characteristics between sample population and total population

Variables	Categories	Sample Percent (%)	Chinese population* Percent (%)
Gender#	Male	49.5	50.6
	Female	50.5	49.4
Age (years)#	<30	26.7	17.6
	30–39	20.4	20.1
	40–49	22.9	18.6
	50–59	14.6	20.0

	≥60	15.4	23.7
Education status†	Junior high school degree or below	22.6	63.0
	Senior high school degree	38.7	18.2
	College degree	19.0	9.7
	Bachelor's degree or above	19.7	9.1
Place of residence‡	Urban	49.3	63.8
	Rural	50.7	36.2

*: based on 2020 national census data from the National Bureau of Statistics

#: based on age ≥ 18 years (Chinese population)

†: based on age ≥ 15 years (Chinese population)

‡: based on age ≥ 20 years (Chinese population)
